# Design of a PEBA–Silicone Composite Magneto-Sensitive Airbag Sensor for Simultaneous Contact Force and Motion Detection

**DOI:** 10.3390/s25185823

**Published:** 2025-09-18

**Authors:** Zhirui Zhao, Chun Xia, Xinyu Zeng, Xinyu Hou, Lina Hao, Dexing Shan, Jiqian Xu

**Affiliations:** 1 School of Mechatronics Engineering, Shenyang Aerospace University, Shenyang 110136, China; douyiming@stu.sau.edu.cn (C.X.); cengxinyu@stu.sau.edu.cn (X.Z.); houxinyu1@stu.sau.edu.cn (X.H.); shandexing@sau.edu.cn (D.S.); xujiqian@sau.edu.cn (J.X.); 2 School of Mechanical and Electronic Engineering, Northeastern University, Shenyang 110819, China; haolina@me.neu.edu.cn

**Keywords:** airbag sensor, contact-force detection, motion detection, PEBA–silicone composite, magneto-sensitive substrate

## Abstract

Considering that soft airbag sensors made from soft materials are limited to detecting only normal forces, a novel PEBA–silicone composite magneto-sensitive airbag sensor is proposed for simultaneously detecting normal contact force and horizontal motion during human–robot interaction. In terms of structural design, the PEBA–silicone composite airbag is manufactured using fused deposition modeling, 3D printing, and silicone casting, achieving a balance between high airtightness and adjustable stiffness. Beneath the airbag, a magneto-sensitive substrate with several NdFeB magnets is embedded, while a fixed Hall sensor detects spatially varying magnetic fields to determine horizontal displacements without contact. The results of contact-force and motion experiments show that the proposed sensor achieves a force resolution of 20 g, a force range of 0 to 1100 g, a fitting sensitivity of 7.54 N/Pa, an average static stiffness of 4.82 N/mm, and a horizontal motion detection range of 0.125 to 1 cm/s. In addition, the prototype of the sensor is lightweight (with the complete assembly weighing 81.25 g and the sensing part weighing 56.13 g) and low-cost, giving it potential application value in exoskeletons and industrial grippers.

## 1. Introduction

Without the rapid development of abundant sensing systems, artificial intelligence techniques, and embodied intelligence systems, robotics may regress to a rigid master–slave architecture bereft of intelligence, activity, or adaptability. In industrial manipulators, integrated force, tactile, and motion sensing transforms traditional open-loop operations into precise, closed-loop control of contact forces, friction, and posture, allowing reliable assembly, compliant grasping, and versatile tool use in uncertain or flexible environments [1,2]. Meanwhile, wearable robots and exoskeletons also rely on force, torque, joint position, and biosensor systems to design human–robot interactive control algorithms that assist users with load support, walking or re-walking, and training upper limbs for passive and active rehabilitation tasks [3,4,5].

Currently, the focus of robotic arms has shifted from rigid single-function industrial applications to safe and compliant human–robot interaction, as well as soft material products that conform to the human body [6,7,8,9,10,11,12,13,14,15,16]. Flexible and soft robots are advancing rapidly, transitioning from lab prototypes to real-world applications in healthcare and exploration [17,18,19]. Soft materials (such as silicone, thermoplastic polyurethane (TPU), and polyether block amide (PEBA)) are conventionally defined as materials with a Young’s modulus below 1 GPa [7]. One of the key capabilities of soft materials lies in their ability to maintain mechanical integrity under repeated folding and bending, as their low elastic modulus imparts intrinsic flexibility without causing permanent damage [8]. Consequently, one of the main applications of these materials is their widespread use in fabricating different kinds of soft grippers for safely and easily grasping delicate and irregularly shaped objects without crushing [9,10]. Additionally, they are commonly used to fabricate soft-bodied biomimetic robots that emulate elephant trunks, octopus tentacles, and peristaltic insects, endowing them with continuous deformation capabilities [11,12,13]. These materials also possess excellent biocompatibility and biodurability, demonstrating their intrinsic compatibility with human limbs in interactions between machines and users [14]. Due to these advantages, soft materials show increasing potential for various wearable robotic systems and smart wearable devices for human–robot interaction tasks. For instance, the silicone-based soft pneumatic elbow pad developed by Xifeng unobtrusively augments the strength of the human elbow joint while maintaining effortless wearability in passive rehabilitation [15]. Mengxin et al. designed a novel polyurethane-based actuator that mimics hierarchical textile architectures, serving as the primary actuator for an upper-limb-wearable soft exoskeleton suitable for medical rehabilitation [11]. Benjamin designed a novel 3D-printed soft pneumatic bending actuator and employed it in rehabilitation gloves, using force and surface electromyography signals to control the glove, allowing the user to easily grasp a water cup [16].

Moreover, most commercially available sensors, such as multi-dimensional force or torque sensors, are difficult to integrate into soft robotic systems because of their rigid structure and high weight. Therefore, the above-mentioned materials have been intensively studied in recent years for application in different types of sensors, especially in force and tactile detection [20,21,22,23,24,25,26,27]. Among these, airbag-type sensors are a promising approach. Their principle is based on changes in the internal air pressure of the airbag in response to contact forces [20]. Since these materials intrinsically conform to large deformations under external force, compression of the compliant airbag walls increases internal air pressure while preventing localized stress on the contact surface. They also enable safe human–robot interaction and can be employed for robot force-control tasks [23]. For instance, Hui et al. fabricated a silicone-based compliant force sensor in the form of an airtight airbag and installed it on the fingers of a pneumatic robot. Loads applied to the airbag substrate changed its internal air pressure, enabling accurate weight measurement [20,21]. Hao et al. developed a silicone airbag sensor using the same structural and measurement principles for an exoskeleton interface supporting human lifting tasks [24]. To prepare an airbag, however, the typical method focuses on silicone pouring into 3D-printed molds (such as polylactic acid (PLA) and acrylonitrile butadiene styrene (ABS)) [20,21,22]. This method introduces internal defects such as parting lines, localized thickness variations, and residual micro-bubbles, which can compromise the uniformity of the elastic modulus, increase nonlinear uncertainty, and render it susceptible to failure under repeated forces [25]. To address these flaws, an attempt has been made to employ soluble mandrels (such as polyvinyl alcohol (PVA) or paraffin wax) instead of PLA or ABS mandrel molds to ensure the integrity of silicone airbags; however, this risks leaving residues. Meanwhile, TPU and PEBA are now common in soft robotics and offer new possibilities for fabricating airbag-based sensors, whose higher elastic modulus theoretically enhances both overall stiffness and linearity [26,27].

However, the airbag-type sensor remains limited in current robotic systems because it operates in only one sensing dimension and rarely captures normal contact force. To address the aforementioned flaws, several multimodal soft tactile sensors, such as piezoelectric tactile sensors, capacitive flexible electronic skin, and soft magnetic skin, have been developed and mounted on robotic fingers to build tactile-force sensing systems [28,29,30,31]. Magnetic skin offers the advantages of low hysteresis, full-surface continuous sensing, and high accuracy, while its structural design can be readily adapted to meet the needs of different application scenarios [31]. For instance, by tailoring the percentage of NdFeB microparticles embedded in the soft, compliant substrate, the magneto-mechanical transduction sensitivity can be significantly enhanced. Upon substrate deformation, the resulting magnetic-field perturbation is instantaneously detected by a Hall sensor or magnetometer, enabling simultaneous quantification of the contact force, indentation depth, and added mass. However, the intricate fabrication process renders low-cost, large-scale production unfeasible. Inspired by the multimodal soft tactile sensing method and the magnetic skin detection principle, this study focuses on a hybrid approach that integrates an airbag with magnetic technology. A PEBA–silicone composite magneto-sensitive airbag contact-force sensor is designed based on a soft material substrate. As a flexible contact-force sensor, the PEBA–silicone composite airbag converts external force into changes in internal air pressure to detect normal contact force, while the magneto-sensitive surface layer is positioned on the top of the airbag to detect horizontal motion. When the surface undergoes horizontal displacement, the movement of the silicone substrate induces a corresponding shift in the alternating arrangement of the north- and south-pole magnets embedded therein. In this case, the externally mounted Hall unit detects changes in the magnetic-field strength and direction. Compared with other airbag sensors, the proposed sensor can simultaneously detect both contact force and horizontal movement. It also inherits the advantages of airbags in terms of low-cost fabrication, lightweight design, and excellent airtight performance.

This paper discusses the fabrication process of a novel airbag contact-force sensor, focusing mainly on a soft PEBA–silicone composite airbag with a magneto-sensitive substrate. Experimental results are presented to verify detection performance for contact force and horizontal movement. Although the proposed sensor and its associated work are not yet fully mature, they have already demonstrated potential application value in physical human–robot interaction (pHRI) within robotic systems [24,32]. The content of this paper is organized as follows. Section 2 presents the structural design methodology and preparation process of the PEBA–silicone composite and the magneto-sensitive substrate for the airbag contact-force sensor, illustrating the principles of the airbag and the magneto-sensitive substrate. Section 3 reveals the characteristics of the sensor prototype system through contact-force and surface-motion experiments. Section 4 provides the conclusions and directions for future work.

## 2. Materials and Methods

**Figure 1:**
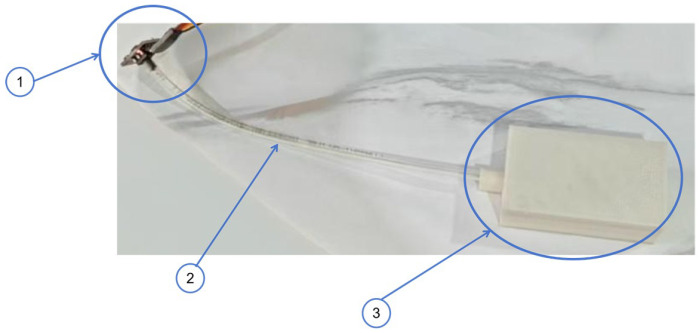
Airbag contact-force sensor: (1) air-pressure sensor; (2) soft air tube; (3) airbag.

In previous studies, airbag-type sensors were composed of airtight airbags, soft air tubes with connectors, and pressure sensors, as shown in Figure 1 [24]. The soft airbag, acting as both a force interface and a sensing unit, converts any force applied to its top surface into a volume change that proportionally alters the internal air pressure, which is detected by the air-pressure sensor through the soft air tube [24]. According to the Clapeyron equation, the deformed volume of the airbag ($V_{2}$) is ideally calculated using the compressed pressure ($P_{2}$), initial air pressure ($P_{1}$), and initial volume ($V_{1}$) as

(1) $$V_{2}=\frac{P_{1}}{P_{2}}V_{1}$$

The structure of the top layer of the airbag is ideally modeled as a simply supported beam, where the relationship between the normal contact force and the air pressure is expressed as [23]

(2) $$F_{c}=K\left(\frac{e^{\prime }l^{\prime }P_{1}}{P_{2}el^{4}}EIV_{1}+el\left(P_{2}-P_{1}\right)\right)$$

where *e* and e′ are the beam widths before and after deformation, *l* and l′ are the corresponding lengths, *E* is the elastic modulus, *I* is the second moment of area, and *K* is a constant structural factor. Since *l* is much larger than *e* and $e^{\prime }$, $\frac{e^{\prime }l^{\prime }P_{1}}{P_{2}el^{4}}\to 0$, the contact force exhibits linearity with the change in air pressure ($P_{2}-P_{1}$) within the airbag, which is ideally similar to Hooke’s law.

(3) $$F_{c}=K_{s}\left(P_{2}-P_{1}\right)$$

where $K_{s}$ denotes the constant coefficient of the sensor, such as sensitivity. However, due to their low stiffness and high elasticity under load, soft materials such as silicone exhibit inherent limitations in the practical applications of the above model.

**Figure 2:**
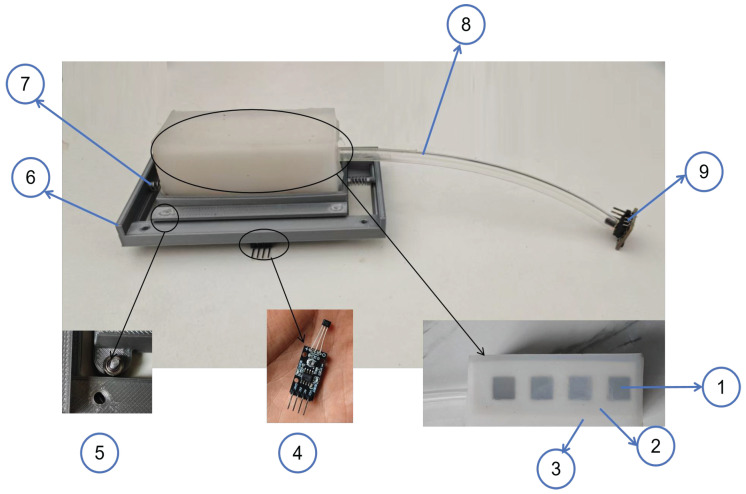
Structure of the magneto-sensitive airbag contact-force sensor: (1) magneto-sensitive substrate; (2) PEBA airbag; (3) silicone shell; (4) Hall sensor; (5) bearing; (6) PLA shell; (7) spring; (8) soft air tube; (9) air-pressure sensor.

Despite the theoretical simplicity of the above-mentioned principle, the measurement accuracy and reliability of the airbag contact-force sensor depend on the structural characteristics of the airbag. Its performance is determined by the geometric dimensions and the integrity of the hermetic seal on the one hand, and by the structural rigidity of the constituent material on the other. Insufficient rigidity of the airbag can lead to non-uniform deformation under load, while poor airtightness of the airbag can cause distortion in pressure transmission. Therefore, this section discusses the fabrication process of airbags and compares the performance of three soft airbags: one made of pure silicone, one of a TPU–silicone composite, and one of a PEBA–silicone composite. The parameters of these materials are listed in Table 1. In addition, this section elucidates the improved design of the magneto-sensitive-substrate airbag contact-force sensor and its preparation process. Unlike traditional airbag sensors, the proposed sensor is referred to as a magneto-sensitive airbag contact-force sensor, whose assembly is shown in Figure 2. The magneto-sensitive layer is placed beneath the airbag, and its surface is embedded with alternating north- and south-pole magnets, causing the magnetic-field direction to flip periodically. Moreover, a Hall sensor is used to measure magnetic-field changes caused by horizontal motion on the silicone surface. Meanwhile, springs and bearings are installed in the PLA shell to ensure smooth horizontal motion without displacement in any other direction when subjected to external forces. The mass of the whole sensor system is only 81.25 g.

### 2.1. Preparation of the Airbags

#### 2.1.1. Silicone Airbag

**Figure 3:**
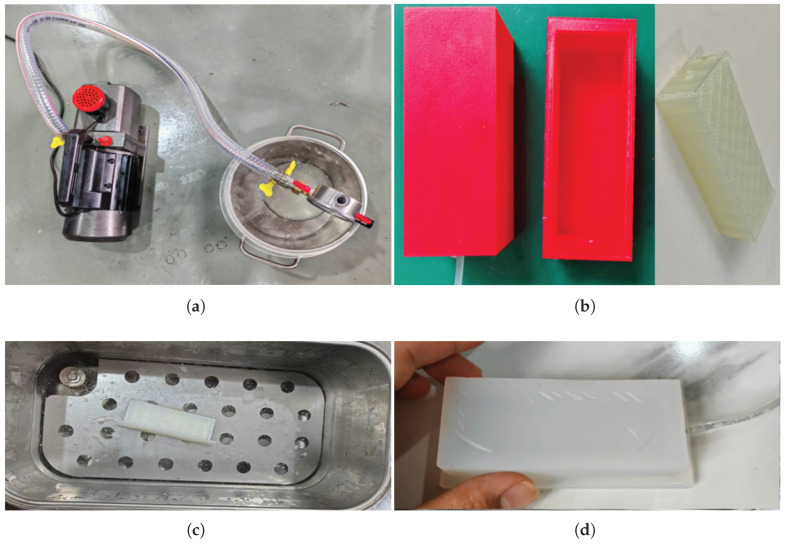
Preparation of the silicone-poured airbag: (**a**) mixing and degassing; (**b**) casting molds; (**c**) dissolving in a water bath; (**d**) final form.

Unlike the other two methods of preparing airbags, silicone-poured airbags involve a pouring process. Studies have shown that paraffin wax or PVA can serve as a filler material in molding, which was adopted in this study [33,34]. Silicone with a Shore hardness of 25A is considered a low-grade material since it strikes a balance between lightweight design and force sensitivity [35]. The preparation method is as follows. First, the mixed silicone was degassed in a vacuum chamber and pump at −1 bar (Figure 3a), and then it was poured into casting molds. The outer mold was made of polylactic acid (PLA), and the inner filler mold was made of PVA, both produced with a 3D printer for fused deposition modeling (H2D, Bambu Lab Inc., Shenzhen, China), as shown in Figure 3b. To dissolve the filler, a water-bath method (100 Celsius, over 12 h) was used to accelerate the dissolution of the PVA material, as shown in Figure 3c. The final prototype is shown in Figure 3d. If necessary, any gaps in the airbag can be filled with a small amount of silicone mixture from the outside. The dimensions of the three airbags are shown in Table 2.

#### 2.1.2. TPU–Silicone Composite and PEBA–Silicone Composite Airbags

As mentioned above, TPU and PEBA are two soft materials commonly used for 3D printing. TPU has gained extensive applications since the 3D printing filament has a wide hardness range of 40A to 95A [35]. Although 40A TPU affords greater sensitivity to contact force, it can cause the printer nozzle to clog or result in structural defects. To balance printing technology and sensing capabilities, 66A TPU (FDM-printed, Yitalong Inc., Changsha, China) was selected for the final airbag.

**Figure 4:**
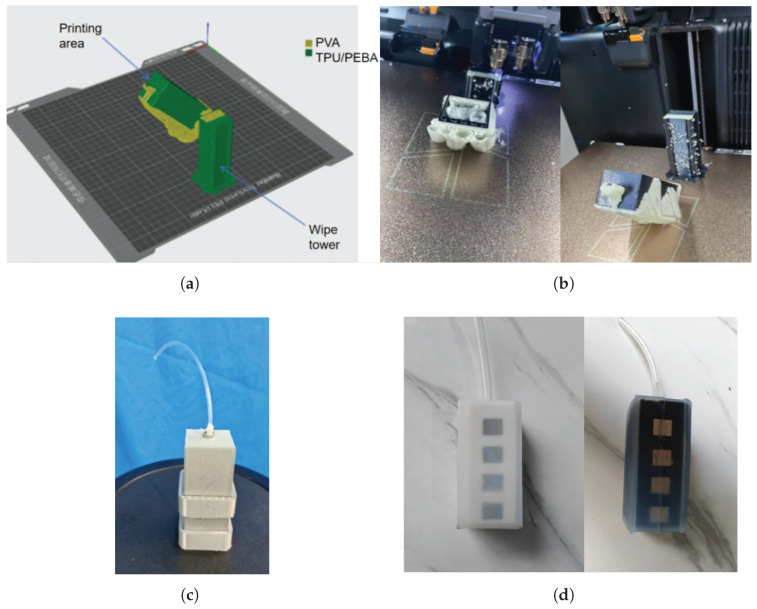
Preparation of the TPU–silicone and PEBA–silicone composites: (**a**) setting the 3D-printing slicer file; (**b**) performing the 3D-printing process; (**c**) pouring the silicone outer shell; (**d**) stripping the mold, mixing, and degassing.

The printing path of the TPU filament employs a concentric path or an Eulerian path, rather than other ones [26]. Afterward, the airbag is designed to withstand external forces in both horizontal and vertical directions, indicating that the connections between the deposited layers of 3D-printed components are the weakest area [36]. This study set a 45-degree inclined printing orientation as the deposition direction. The proposed orientation allows interlayer bonding surfaces to simultaneously share horizontal and vertical stresses, thereby effectively enhancing the overall stiffness of the structure under loading conditions. In addition, during the printing process of soft materials, it is essential to provide both external and internal supports for the airbag, as shown in Figure 4a. In this study, the support material was PVA, which is water-soluble. Since two different materials were used, a wipe tower was employed to purge any residual filament when switching between two 0.6 mm nozzles. The printing process of the TPU airbag is shown in Figure 4b. The temperature was set to 245 degrees Celsius (5 degrees Celsius higher than the melting point) on one printer nozzle for TPU and 210 degrees Celsius on the other nozzle for PVA. Both printing materials were dried in the automatic material system (AMS-HT, Bambu Lab Inc., Shenzhen, China) at 70 Celsius for 8 h, generating a humidity level of 10% to 20% before printing. The other printing parameters were as follows: print speed of 50 mm/s, build-plate temperature of 50 degrees Celsius, close-and-withdrawal action, TPU infill density of 100%, and PVA infill density of 15%.

Meanwhile, due to the low 3D-printing speed of TPU, PEBA, another soft material used in 3D printing with a typical hardness range of 80A to 95A for its filaments (Xinbochuan Advanced Materials Technology Co., Shenzhen, China) was selected as an alternative. It offers improved durability through its superior elasticity and abrasion resistance, thereby extending the product’s lifespan. A PEBA airbag was then printed using the FDM 3D-printing method, with the remaining settings and path selections as mentioned above, in addition to a temperature setting on the printer nozzle of 250 degrees Celsius (5 degrees Celsius higher than the melting point). The printing speed was set at 100 mm/s. To counteract the springback of PEBA, the filament was extruded midair into the feed port of the 3D printer. Since the high elasticity of PEBA can cause rebound at the feed inlet, the printing process was continuously monitored, and the withdrawal distance was set to 2 mm in the 3D-printing software. After printing, the magneto-sensitive substrate of the sensor was prepared, as detailed in the next subsection.

However, the low thermal conductivity of soft TPU and PEBA can cause the melting temperature of the freshly extruded layer to drop rapidly below the remelting threshold of the previous layer as heat diffuses into the cooler substrate, resulting in poor airtightness of the printed TPU and PEBA airbags. Even if the printing speed is reduced at the expense of throughput, the problem of insufficient interlayer fusion can persist, producing interfacial defects such as micropores, which ultimately compromise airtightness, particularly in printed airbags. Moreover, the high resilience of PEBA and the moisture absorption of TPU during the feeding process can interrupt the supply of the printing filament for 3D printers in unpredictable ways. To avoid these issues, this study used a casting process to form a Shore 25A silicone shell over the 3D-printed airbag. As illustrated in Figure 4c, a PLA mold was employed to create a conformal silicone shell. The final TPU–silicone composite and PEBA–silicone composite airbags are shown in Figure 4d.

**Figure 5:**
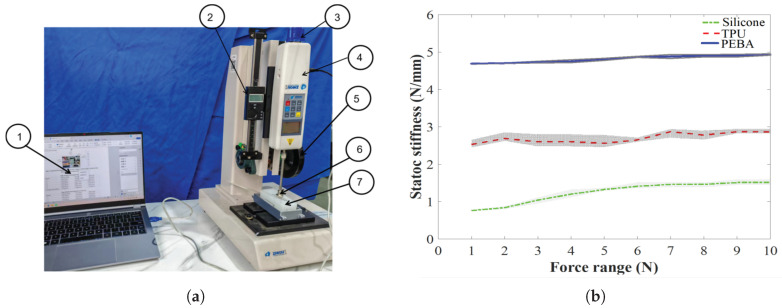
Static stiffness experiment. (**a**) Experimental setup: (1) laptop; (2) vernier caliper; (3) handwheel; (4) RS232-to-USB data cable; (5) force measurement device; (6) force probe; (7) proposed sensor. (**b**) Static stiffness curves.

Subsequently, the static stiffness values of the three airbags were evaluated. The experimental setup mainly included a force measurement device (Precision Instruments Hp50, ZHIQU Inc., Dongguan, China), a laptop (CPU: r7-8845h; RAM: 24 GB), and a vernier caliper (resolution: 0.01 mm), as shown in Figure 5. The measurement device was used to measure the contact force applied to the airbag, allowing manual adjustment to control the descent or ascent of the force probe using a handwheel, while the contact force between the airbag and the probe was transmitted to the laptop via an RS232-to-USB cable. Meanwhile, a vernier caliper was used to record the deformation displacement of the airbag’s top surface in a stable state. During the experiment, an external load was applied incrementally, starting from 0 N and increasing by 1 N at each step until reaching a maximum force of 10 N. The measurement data was collected at intervals, as shown in Figure 5. Each group of measurements was spaced more than 30 s apart, and each data point in the three curves represents the mean of five trials to minimize operational and experimental errors. The results indicated that, owing to its inherently low material stiffness, the silicone airbag exhibited the lowest static stiffness (0.76 N/mm to 1.51 N/mm), followed by the TPU–silicone composite airbag (2.53 N/mm to 2.87 N/mm) and the PEBA–silicone composite airbag (4.69 N/mm to 4.93 N/mm). Meanwhile, the average stiffness values of the three materials were substituted into the following equation to calculate the variance. Analysis of variance quantifies the within-group variability of each data point, allowing us to assess how consistently the stiffness coefficient is preserved across the three airbags. A lower variance indicates a more linear, stable stiffness for that airbag material.

(4) $$Var\left(x,\bar{x},n\right)=\sqrt{\sum {\left(x-\bar{x}\right)}^{2}/n}$$

where $\bar{x}$ denotes the average value of *x* and *n* denotes the number of the measurement.

From the calculations, the following observations were made:

1. The first five stiffness measurements of the pure silicone airbag were all below its average stiffness of 1.25 N/mm, reflecting pronounced nonlinearity.
2. Owing to its greater hardness, the variance of the PEBA–silicone composite exhibited a lower value than that of the TPU–silicone composite airbag and the pure silicone airbag (0.0059 < 0.0141 < 0.0744). Meanwhile, the PEBA–silicone composite airbag achieved the best average stiffness (4.82 N/mm) and retention capacity among the three airbags under loads from 0 to 10 N.

### 2.2. The Magneto-Sensitive Substrate

**Figure 6:**
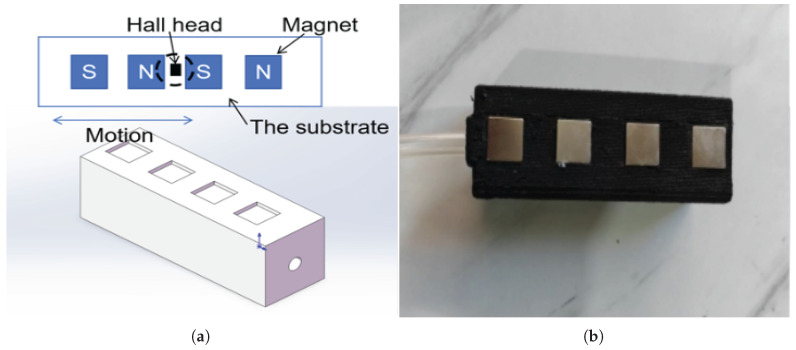
Preparation of the magneto-sensitive substrate: (**a**) basic principle; (**b**) final form.

The magneto-sensitive substrate is another main component of the proposed sensor. Taking the upper-limb exoskeleton for active rehabilitation as an example, the external force applied by the wearer on the human–robot interface is not purely normal force on the top surface of the airbag but is typically accompanied by some tendencies of horizontal motion. Therefore, the proposed sensor focuses on detecting motion information rather than contact force alone. The principle of the proposed sensor was inspired by magnetic-scale technology, which detects displacement in non-contact ways by exploiting variations in the magnetic field. It consists of a substrate (positioned in the bottom layer of the airbag) embedded with evenly spaced NdFeB magnet poles (10 mm × 10 mm × 1 mm, magnetic field ranging from 400 to 500 G), whose north and south polarities alternate upward, and a stationary Hall sensor that lies parallel to the substrate and acts as a reading head, as shown in Figure 6. With the magneto-sensitive substrate embedded in the airbag, the Hall sensor (Y315Hall, Yunou Inc., Changsha, China, 23 kHz sampling frequency, ±1500 G measurement range) detects variations in the magnetic field caused by its motion. Because permanent magnets alternate between the north and south poles at equal intervals, the direction of motion can also be determined. Dedicated equipment, such as a Gaussmeter, can eliminate the above delays but introduces additional costs and sensor bulk to the application.

The fabrication of the magneto-sensitive substrate comprised three main steps. First, the substrate structure was produced by 3D printing, which could be carried out concurrently with the fabrication of the TPU/PEBA airbag, as shown in Figure 4b. Second, the magnets were embedded in the substrate with cyanoacrylate adhesive, ensuring that adjacent magnets did not attract each other. Finally, the outer shell of the magneto-sensitive substrate was cast in silicone Shore 25A using the PLA mold shown in Figure 4c.

## 3. Experiments and Results

In this section, the proposed sensor is assembled and subjected to a comprehensive experimental campaign. The evaluation proceeds in two parts. First, we systematically examine the contact-force measurement performance of the three candidate airbag materials, yielding quantitative estimates of system stiffness (Equation (1)). Second, we characterize the motion-detection capabilities of the sensors. The results substantiate that the proposed sensor surpasses conventional airbag-based counterparts by furnishing substantially richer information, which holds significant potential in human–robot interaction scenarios involving exoskeletons.

### 3.1. Contact-Force Experiment

**Figure 7:**
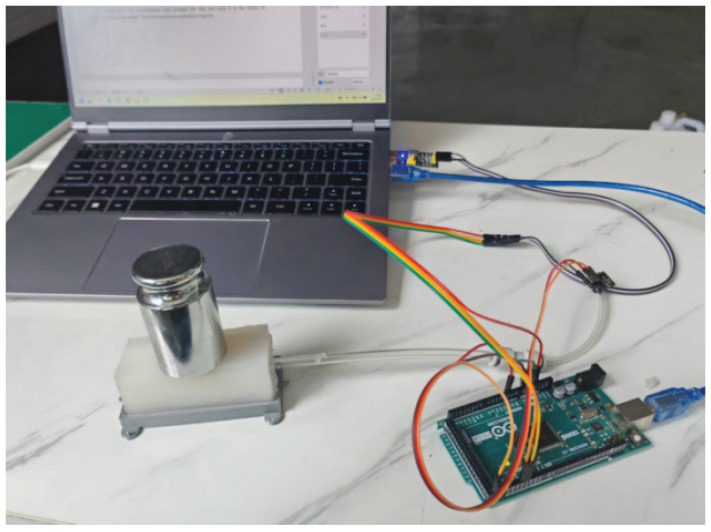
Experimental setup (contact-force experiment).

The experimental setup comprised a series of standard calibration masses (weighing 10 g to 1 kg, Xingling Precision Instrument, Shenzhen, China), an air-pressure sensor (XGZP6847A, CFSensor Ltd., Wuhu, China), a microcontroller (Arduino Mega 2560, Arduino LLC, Turin, Italy), a USB-to-serial adapter, and a laptop. The sensor prototype was placed horizontally on a laboratory bench in a stable configuration, as shown in Figure 7. Several calibration masses were applied to the proposed sensors. The air-pressure sensor detected the difference in the internal pressure of the airbag after a contact force was applied and transmitted these values to the microcontroller through its analog pins. The microcontroller then packaged the data and sent it to the laptop via a USB-to-serial adapter.

**Figure 8:**
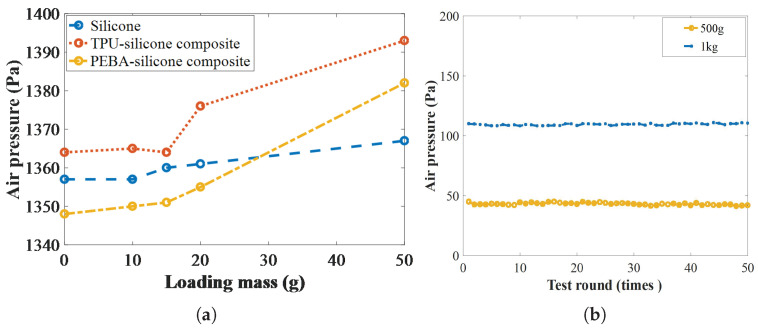
Resolution and repeatability of the proposed sensor: (**a**) resolution test; (**b**) repeated loading test.

First, the normal-force resolution of the proposed sensor was experimentally characterized with small-mass weights of 5, 10, 20, and 50 g. To eliminate measurement errors, five test groups were conducted; in each group, the airbag was measured five times under controlled conditions (28–30 Celsius, 40–45% RH). During the test process, the weights were placed successively at the center of the airbag, from the smallest to the largest, while the pressure sensor monitored the minimum load that resulted in a discernible change in internal pressure. The results in Figure 8 show that all three airbag designs achieved a resolution of 20 g. Pure silicone showed the highest sensitivity (a resolution of 15 g) due to its relatively soft Shore hardness, whereas the TPU–silicone and PEBA–silicone composite airbags both achieved 20 g.

To demonstrate the repeatability of the proposed sensor, 50 trials at 500 g and another 50 trials at 1 kg were carried out. The PEBA–silicone composite airbag rested on a stable bench at 28 to 30 Celsius, with the load centered on its top; no air leakage was observed after 100 consecutive tests. The difference between the internal air pressure and the initial value was recorded, as shown in Figure 8. The data showed variances of 1.149 (1 kg) and 0.483 (500 g), and the largest deviations from the respective means were 1.542 and 1.574, confirming that the sensor provides stable and reliable measurements over the repeated loading test.

**Figure 9:**
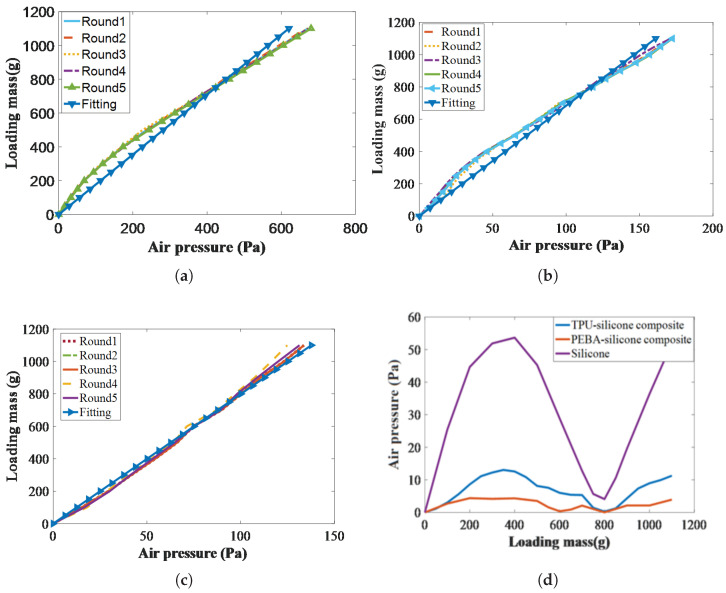
Results of the contact-force experiment: (**a**) silicone; (**b**) TPU–silicone composite; (**c**) PEBA–silicone composite; (**d**) fitting errors under different loading masses.

Afterward, to characterize the relationship between the contact force and the response air pressure, each round started after confirming airtightness and applying the same normal contact-force range of 0 to 1100 g to all three airbags in increments of 50 g. Five rounds of experiments were conducted on each airbag, and the average measurement values were calculated, as shown in Figure 9a–c. The abscissa represents the air-pressure difference, calculated as the current pressure minus the initial pressure. The above force range corresponds to the 0 to 10 N safety threshold for human–robot interaction tasks in references [37,38]. Consequently, the linearity of the constant coefficients of the sensor $K_{s}$ (in Equation (3)) was completely different. Obviously, the silicone airbag exhibited a nonlinear trend between the air pressure and contact force within the range of 0 to 450 g, which is consistent with the earlier stiffness characterization. However, as shown in Figure 9b, the experiments also revealed that the TPU–silicone composite airbag exhibited nonlinear behavior, with two distinct stiffness regimes between 300 and 350 g that differed from other ranges. However, the PEBA–silicone composite airbag exhibited a markedly linear stiffness across the entire force range, indicating that the adhesive silicone layer exerted no noticeable influence due to the superior static stiffness of the other two soft materials (2.82 N/mm > 1.70 N/mm > 1.25 N/mm).

Additionally, the $K_{s}$ values were calculated using the fitted curves of the pure silicone, TPU–silicone composite, and PEBA–silicone composite prototypes, yielding 1.76 N/Pa (R-square = 0.92, RMSE = 11.81), 6.72 N/Pa (R-square = 0.97, RMSE = 9.61), and 7.54 N/Pa (R-square = 0.99, RMSE = 2.49), respectively. Then, the absolute errors were plotted with the loading mass as the abscissa and the air-pressure difference as the ordinate, as shown in Figure 9d. This figure clearly demonstrates that the PEBA–silicone composite prototype achieved significantly higher accuracy than both the TPU–silicone composite and pure silicone prototypes. In terms of maximum absolute error, the PEBA–silicone composite registers 4.81 Pa, lower than the TPU–silicone composite at 11.35 bar and far below the pure silicone prototype at 56.78 Pa.

### 3.2. Motion Detection

**Figure 10:**
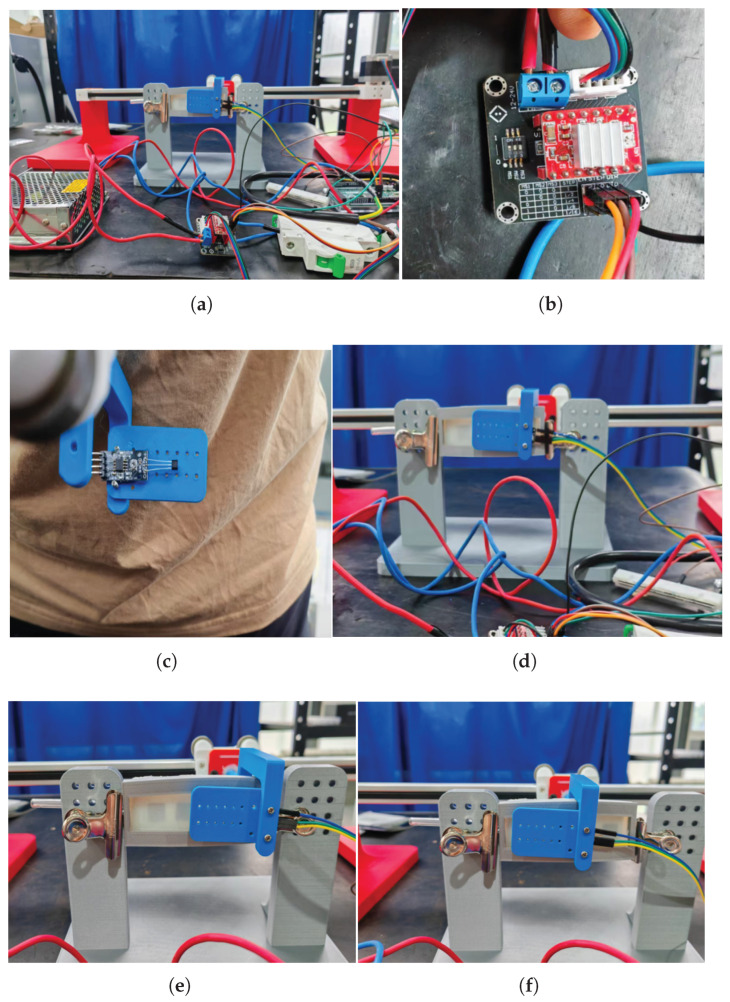
Surface-motion experiment: (**a**) experimental setup; (**b**) driven PCB card; (**c**) Hall sensor; (**d**) initial statement; (**e**) leftward movement; (**f**) rightward movement.

In this subsection, the motion-detection capabilities of the proposed sensor are verified through an experiment designed to mimic the motion process during human–robot interaction, as shown in Figure 10a. The setup involved a 500 mm horizontal, low-friction sliding support driven by a 42-stepper motor, creating the required relative motion between the proposed sensor and a Hall sensor. A driver PCB card (A4988, Mixly Inc., Shaoxing, China) was used to control the stepper motor (Figure 10b). The Hall sensor was fixed on the sliding support, and a 3D-printed frame mounted the proposed sensor (Figure 10c). Subsequently, the microcontroller acquired measurement data, and the laptop recorded the data through the USB-TTL serial port. The experiment mainly evaluated the ability to determine motion direction and the bandwidth for recognizing motion speed.

The experiment proceeded as follows: first, the horizontal low-friction sliding stage was homed so that the Hall probe sat midway between two permanent magnets; second, through a USB-TTL serial port, the host PC sent a motion command to the microcontroller, which output PWM to the driver board to generate the controlled relative displacement between the magnets and Hall sensor; and finally, multiple runs were conducted by alternately reversing the stage direction (through the direction pin of the driver PCB card set as high or low) and adjusting the speed (by setting the stepper motor microstepping to 1, 1/2, 1/4, and 1/8), as shown in Figure 10d–f. The above process was divided into eight groups: groups 1 to 8 corresponded to leftward movement at standard speed, rightward movement at standard speed (about 1 cm/s), leftward movement at half-standard speed, rightward movement at half-standard speed, leftward movement at one-quarter standard speed, rightward movement at one-quarter standard speed, leftward movement at one-eighth standard speed, and rightward movement at one-eighth standard speed, respectively. The data collected from the Hall sensor and the step count of the motor were recorded by the microcontroller and continuously transmitted to the laptop. During the experiment, data from the Hall sensor was collected at a frequency of 10 Hz.

**Figure 11:**
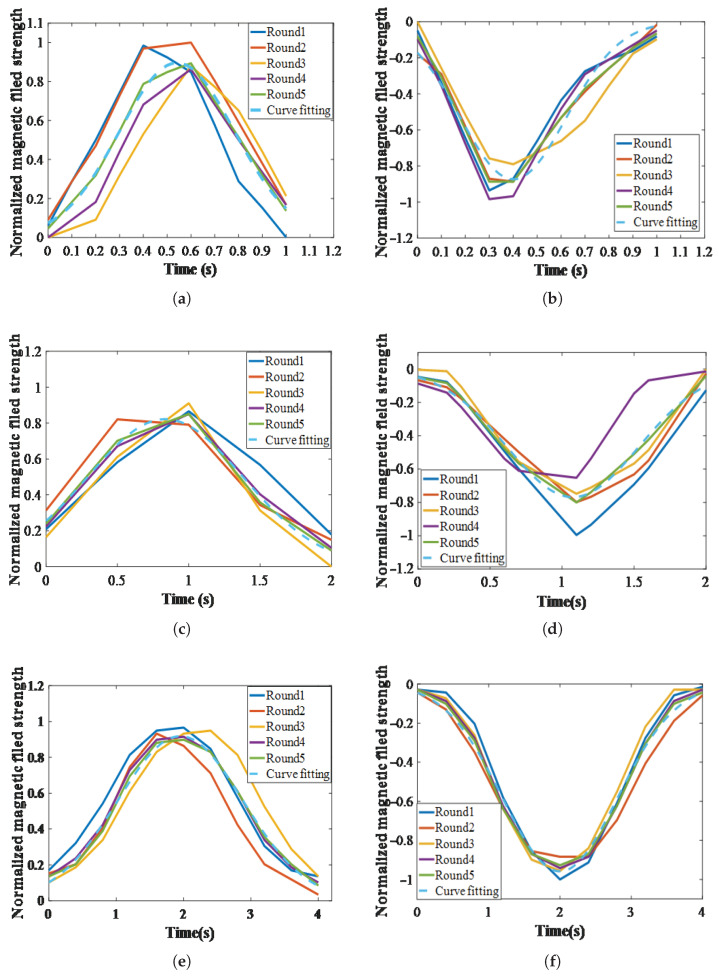

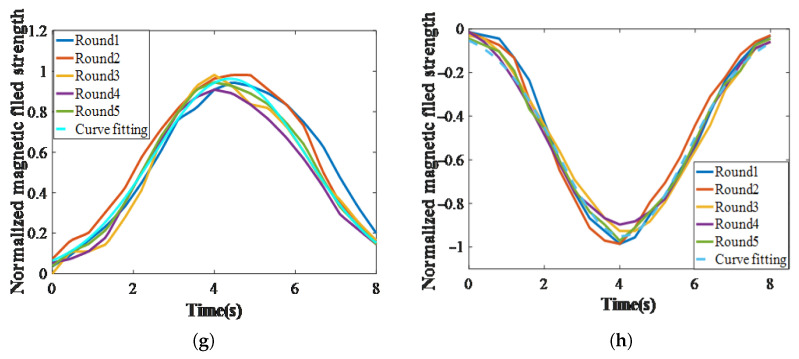
Curves of the normalized magnetic-field strength: (**a**,**b**) at a speed of 1 cm/s. (**c**,**d**) at a speed of 0.5 cm/s. (**e**,**f**) at a speed of 0.25 cm/s. (**g**,**h**) at a speed of 0.125 cm/s.

Specifically, each group of motion consisted of a unidirectional linear motion over a fixed travel distance of 20 mm spanning one permanent-magnet width. When the laptop sent a left-shift command to the microcontroller, the Hall sensor slid over a north-pole magnet in the middle of the substrate and stopped in the gap between two adjacent magnets, whereas a right-shift command passed over the south-pole magnet. Consequently, the two motions generated entirely different waveforms. The magnetic flux density acquired by the Hall sensor was normalized using the normalization equation (Equation (5)), yielding the waveform shown in Figure 11. As the prescribed velocity decreased, the curve of the normalized magnetic-field strength increasingly approximated the Gaussian function in Equation (6). The fitting parameters and the correlation coefficient r are shown in Table 3. Specifically, at speeds of 1, 0.5, 0.25, and 0.125 cm/s, the value of r monotonically increased as the speed decreased. When the speed dropped to 0.25 cm/s or lower, the correlation became stronger (r > 0.95), indicating that the sensor is suitable for fine displacement measurements under such conditions.

Moreover, the motion direction can be distinguished by the field orientation (i.e., positive means leftward and negative means rightward). The above results demonstrate that the proposed sensor responds effectively to motion velocities from 0.125 cm/s to 1 cm/s. Detection clarity increases as the motion slows down. It should also be noted that, at high motion speeds (1 cm/s), the Hall sensor exhibits a delay phenomenon when measuring magnetic-field strength.

(5) $$N_{i}=\frac{G_{i}-G_{\min}}{G_{\max}-G_{\min}}$$

where $G_{i}$, $G_{min}$, and $G_{max}$ denote the ith value, the minimum, and the maximum of the vector G, respectively.

(6) $$f\left(t\right)=a_{1}\exp\left({\left(-\left(t-b_{1}\right)/c_{1}\right)}^{2}\right)$$

where $a_{1}$, $b_{1}$, and $c_{1}$ denote the parameters of the Gaussian function.

## 4. Conclusions

In this study, a novel magneto-sensitive airbag sensor is proposed to simultaneously detect the normal contact force on the top layer of the airbag and its horizontal movement. The principles of 3D-printed airbags and magnetic substrates, as well as the sensor preparation steps, are discussed. Moreover, the performance of the proposed sensor is verified through contact-force and motion-detection experiments, achieving a force resolution of 20 g, a force range of 0 to 1100 g, and a horizontal motion range of 0.125 to 1 cm/s. Although the sensor still falls short in body-size sensing, dynamic performance, real-time sensing, and force-direction perception, its low cost and multimodal capabilities demonstrate its significant potential for human–robot interaction environments. Future efforts will focus on optimizing the structural dimensions and performance metrics of the proposed sensor to establish accuracy models, develop more efficient fabrication processes for soft materials, and integrate sensors into exoskeletons and industrial robot grippers for practical applications.

## Figures and Tables

**Table 1:** Parameters of the soft materials used in this study.

Material	Hardness	Density	Elastic Modulus	Melting Point
Silicone	25A	1.1 g/${\mathrm{cm}}^{3}$	0.8 to 1.2 MPa	Not given
TPU	66A	0.54 g/${\mathrm{cm}}^{3}$	3.59 MPa	240 Celsius
PEBA	80A	0.8 g/${\mathrm{cm}}^{3}$	9.65 MPa	245 Celsius

**Table 2:** Dimensions of the three airbags.

Material	Length	Width	Height	Thickness	Mass
Silicone	85 mm	30 mm	25 mm	3 mm	74.77 g
TPU–silicone composite	82 mm	28 mm	25 mm	1.5 mm TPU and 2 mm silicone	53.12 g
PEBA–silicone composite	82 mm	28 mm	26 mm	1.5 mm PEBA and 2 mm silicone	56.31 g

**Table 3:** Curve-fitting parameters.

Vector	$a_{1}$ (Left/Right)	$b_{1}$ (Left/Right)	$c_{1}$ (Left/Right)	*r* (Left/Right)
1 cm/s	0.8962/−0.8741	0.5414/0.4005	0.3422/0.3141	0.83/0.89
0.5 cm/s	0.8217/−0.7683	1.075/0.8358	0.6479/0.7728	0.91/0.87
0.25 cm/s	0.918/−0.9587	2/1.952	1.144/1.319	0.95/0.97
0.125 cm/s	0.9625/−0.9496	4.068/4.382	2.399/2.667	0.99/0.99

## Data Availability

Data is contained within the article.

## Abbreviations

The following abbreviations are used in this manuscript:

3D	Three-Dimensional
PEBA	Polyether Block Amide
TPU	Thermoplastic Polyurethane
PLA	Polylactic Acid
PVA	Polyvinyl Alcohol
